# Total iron binding capacity (TIBC) is a potential biomarker of left ventricular remodelling for patients with iron deficiency anaemia

**DOI:** 10.1186/s12872-019-01320-3

**Published:** 2020-01-08

**Authors:** Yan Chen, Jing Wan, Haidan Xia, Ya Li, Yufeng Xu, Haiyan Lin, Hassah Iftikhar

**Affiliations:** 1 Division of Cardiology, Department of Medicine, Zhongnan Hospital, Wuhan University, Wuhan, Hubei People’s Republic of China 430071; 2 Division of Hematology, Department of Medicine, Zhongnan Hospital, Wuhan University, Wuhan, Hubei People’s Republic of China 430071

**Keywords:** Total iron binding capacity, Left ventricular mass index, Iron metabolism, Iron deficiency anaemia, Cardiac remodelling

## Abstract

**Background:**

Preclinical studies indicate iron deficiency (ID) plays an important role in cardiac remodelling. However, the relationship between ID and cardiac remodelling remains unknown in clinical setting. This retrospective study aims to identify a potential biomarker for the myocardial remodelling in patients with ID. Due to limited patients with ID are identified without iron deficiency anaemia (IDA), we analyse the relationship of total iron binding capacity (TIBC) and the left ventricular mass index (LVMI) in patients with iron deficiency anaemia.

**Methods:**

A total of 82 patients with IDA exhibiting the diagnostic criteria for IDA were enrolled in the study. Among the patients, 65 had reported LVMI values. Subsequently, these patients were divided into two groups according to abnormal LVMI (> 115 g/m^2^ in men and > 95 g/m^2^ in women). Linear bivariate analysis was performed to detect the associations of haemoglobin or TIBC with clinical and echocardiographic characteristics. Simple linear regression analysis was used to evaluate the correlation between LVMI and the parameters of IDA, while multivariable linear analysis was used to assess the association of LVMI with age, TIBC and haemoglobin. Logistic regression analysis was utilized to determine the relationship of LV remodelling with anaemia severity and TIBC.

**Results:**

As compared with control group, the levels of TIBC in abnormal LVMI group are increased. Using log transformed LVMI as the dependent variable, simultaneously introducing age, TIBC, and haemoglobin into the simple linear regression or multivariable linear regression analysis confirmed the positive association among these factors. Bivariate correlation analysis reveals the irrelevance between haemoglobin and TIBC. In logistic regression analysis, TIBC is associated with the risk of LV remodelling.

**Conclusions:**

Results of study indicate that TIBC exhibit an explicit association with LVMI in patients with iron deficiency anaemia. Logistic analysis further confirms the contribution of TIBC to abnormal LVMI incidence among this population with IDA.

## Background

Iron deficiency anaemia (IDA) affects millions of people worldwide, and children and premenopausal women account for a large portion of patients with IDA [1]. In addition, the majority of individuals with iron deficiency (ID) in the early stage of IDA remain undiagnosed in outpatient clinic, and only a few patients reach the point of hospitalization. Because of the frequency of asymptomatic individuals and those with only subtle signs, the pathophysiological damage induced by the overall time interval of ID is difficult to trace and can merely be discovered after time-delayed manifestation [2]. Thus, the potential hazard underlying ID may be underestimated.

Cardiovascular manifestations can be observed in patients with IDA and in those with sickle cell disease (SCD), which indicates ventricular systolic and diastolic dysfunction, elevated cardiac output and cardiomegaly [3–5]. Iron metabolism is also inextricably linked to cardiomyopathy [6]. It has been demonstrated that cardiomyopathy is associated with iron overload, and the production of hydroxyl radicals and lipid peroxidation have been shown to play key roles in the initiation of cardiomyopathy [7, 8]. However, the inverse interaction between ID and cardiac cells is indefinite. It has reported that malnutrition-induced ID in growing rats leads to cardiomyopathy [9], and a recent study also showed that mice with transferrin receptor knockout in the heart may suffer from lethal cardiomyopathy involving impaired mitochondrial biogenesis [10]. Although preclinical study indicates that ID induces cardiomyopathy, [11] clinical evidences are lacking. Moreover, attempts to link myocardial function to ID parameters also *resulted in* failure, [12, 13] and the precise role of iron in human myocardium remains vague. Related hypotheses include oxidative metabolism, cellular immune mechanisms [14] and mitochondrial injury [8, 15].

ID is commonly found in individuals with chronic conditions such as heart failure (HF) and chronic kidney disease (CKD), which concomitantly complicate the pathological interactions among these conditions [14]. Impaired iron metabolism secondary to inflammatory processes together with depletion of iron stores are responsible for ID under above pathological conditions. In addition, ID was proven to be independently related to cardiomyopathy and cardiovascular mortality in these patients, and these conditions could be significantly ameliorated if iron is replenished in time [16]. Thus, we simply excluded these patients considering the excessive heterogeneity they may contribute to the sample and the different age groups to which they belong. Despite the different pathologic mechanisms underlying concurrent ID and ID alone in the general population, traditional ID parameters, such as ferritin and TS, both provide the versatility of diagnosis and treatment guiding [17, 18].

This study aims to further expand this emerging field by revealing ID parameters linked to myocardial remodelling in patients, specifically those with IDA. We hypothesize that ID is correlated with left ventricular remodelling in IDA patients and potential ID indicators are existed to reflect such relationship.

## Methods

### Study population

The 82 enrolled patients were selected from the Devision of Haematology at the Zhongnan Hospital of Wuhan University from March 2013 to June 2018. All patients met the diagnostic criteria for IDA, which referred to the 2007 WHO guidelines for assessing iron status. The additional inclusion criteria were as follows: the duration of symptoms estimated to be more than 3 months; no history of smoking or alcohol use; absence of infection or other comorbidities; and no regular or effective treatment prior to admission. Sixty-five of the enrolled patients were assessed for anthropometric features to determine LVMI.

### Measurements

The duration of symptoms and anthropometric features were acquired from the patient’s medical history or at a follow-up visit, and the duration of symptoms was considered as the approximate iron deficiency duration. Heart rate, blood pressure and blood samples for laboratory assays were obtained at the initial in-hospital visit immediately following the echocardiography assessment.

Venous blood was obtained from each patient at 8:00 a.m. All biochemical indicators were assayed in the central laboratory of the Zhongnan Hospital. Complete blood count was analysed using a UniCel DxH 800 Coulter Cellular Analysis System (Beckman Coulter, Inc., California, USA). Serum ferritin levels were measured by a DxI 600 immunoassay analyser (Beckman Coulter, Inc.). Serum iron and UIBC were measured by AU5810 Chemistry Analyzers (Beckman Coulter, Inc.). TIBC was calculated by adding the value of Fe to that of UIBC.

Two-Dimensional TEE (AUCSON SC2000 echocardiography system, Siemens, Germany) was performed by trained and certified operators according to the standard operating procedures. M-mode measurements yielded left ventricular end-diastolic (LVDd) and end-systolic (LVDs) dimensions, interventricular septum diastolic diameters (IVSTd), and left ventricular posterior wall thicknesses (LVPWT). Both SV and EF were derived from LVDd and LVDs. LVM was calculated using the Devereux formula.

### Statistical analysis

SPSS 22 (IBM SPSS Statistics for Windows, Version 22.0. Armonk, NY, USA: IBM Corp.) was used for statistical analysis. Categorical variables are presented as percentages, while continuous variables are presented as the mean ± SD or the median (interquartile range) without a Gaussian distribution. Because of the non-normal distribution of LVMI in this study, log-transformation was performed when needed. For the 65 patients whose LVMI values were assessed, linear bivariate analysis with Pearson’s correlation coefficient was used to detect associations between age, BMI, HR, haemoglobin, TIBC, EF% and log transformed LVMI. Linear bivariate analysis by Spearman’s rank correlation was used to detect associations among SBP, DBP, serum iron, serum ferritin, TS, LVDd, LVDs, SV, albumin, haemoglobin, TIBC, and LVMI. In the 82 patients, the relationships between haemoglobin, TIBC and clinical and echocardiographic characteristics were explored in the same way.

The group of 65 patients was divided into two groups according to abnormal LVMI (> 115 g/m^2^ in men and > 95 g/m^2^ in women). The characteristics of the two groups were compared using the chi-square test for categorical variables; continuous variables were compared by Student’s t-test or a nonparametric Mann-Whitney U test for non-normally distributed data. Simple linear regression analysis was used to detect associations between log transformed LVMI and age, HR, SBP, DBP, haemoglobin, serum iron, serum ferritin, TIBC, TS, RDW, and albumin. Stepwise multivariable linear regression analysis was used to evaluate the association of log transformed LVMI (dependent variable) with age, BMI, haemoglobin, serum iron, serum ferritin, TIBC, TS, and albumin. The interaction term was added into established multivariable linear regression model to test the interaction. Finally, logistic regression was conducted to measure the associations between TIBC, haemoglobin, age, gender duration of ID and prevalence of LV remodelling. Variables with more than 5% missing data were excluded from the analysis. For variables with less than 5% missing data, mean imputations were used when necessary. All statistical tests were two-tailed, and a *P* value < 0.05 was considered statistically significant. In multiple comparisons section, Bonferroni correction was applied for adjustment of *P* values. Q values of Table 1 are calculated by package “fdrtool” of R (version 3.6.1) software.

**Table 1:** Clinical and echocardiographic characteristics

	Total		LVMI (g/m^2^)		P	Q
	*N* = 82	*N* = 65	Normal (*N* = 42)	Abnormal (*N* = 24)		
Demographic characteristics						
Sex (female) n (%)	65(79.3)	52(80.0)	34(82.9)	18(75)	0.441	0.387
Age (y)	41.67 ± 14.14	42.55 ± 13.36	40.81 ± 13.49	45.54 ± 12.86	0.17	0.273
Height (m)		1.63 ± 0.06	1.63 ± 0.06	1.62 ± 0.08	0.908	0.562
Weight (kg)	57.38 ± 7.25	57.09 ± 7.01	57.60 ± 6.71	56.23 ± 7.56	0.452	0.389
BMI (kg/m^2^)		21.59 ± 2.76	21.72 ± 2.42	21.37 ± 3.32	0.617	0.465
Duration (y)	0.5(2.75)	0.50(2.75)	0.50(1.75)	0.75(8)	0.231	0.312
HR (bpm)	81.24 ± 13.37	81.57 ± 12.98	80.51 ± 12.01	83.38 ± 14.57	0.359	0.365
SBP (mmHg)	120(12)	118(12)	116(11.5)	119(11)	0.29	0.34
DBP (mmHg)	67.5(10.25)	65(12)	65(14)	65.5(7.75)	0.738	0.51
Treatment						
Oral iron	10(12)	6(9.2)	5(12.2)	1(4.2)	0.4	0.377
Intravenous iron	2(2.4)	2(3.1)	1(2.4)	1(4.2)	1	0.585
Blood transfusion	5(6)	5(7.7)	3(7.3)	2(8.3)	1	0.585
Haematologic Parameters						
Hgb (g/L)	60.69 ± 14.54	59.71 ± 14.04	62.03 ± 14.14	55.76 ± 13.24	0.083	0.182
HCT (%)	20.98 ± 4.28	20.86 ± 4.16	21.23 ± 4.37	20.22 ± 3.80	0.351	0.362
MCV (fL)	65.2 ± 9.12	64.98 ± 9.34	65.20 ± 9.33	64.61 ± 9.54	0.81	0.533
RDW (%)	19.9(3.3)	20.2(3.3)	19.4 (3.6)	20.45 (3.1)	0.09	0.191
Iron Parameters						
Serum iron (μmol/l)	4.05 (3.5)	4.1 (3.1)	4.0 (2.8)	4.45 (4.5)	0.995	0.584
Serum ferritin (ng/ml)	4.26 (4)	4.08 (4)	4.12 (5)	3.73 (4)	0.422	0.382
TIBC (μmol/l)	77.7 ± 16.28	75.27 ± 15.98	71.85 ± 14.24	81.12 ± 17.35	0.023	0.067
TS (%)	5.65 (5.79)	5.87 (5.23)	5.87 (5.01)	6.02 (6.43)	0.505	0.416
Ultrasound Parameters						
LVDd (mm)	46.03 (5.92)	45.93 (5.37)	44.90 (3.83)	49.04 (6.85)	< 0.001	0.001
LVDs (mm)	28.13 (5.33)	28.13 (5.33)	27.34 (3.85)	31.19 (5.52)	0.001	0.004
IVSTd (mm)	9 (1)	9 (1)	9 (1)	10 (1.8)	0.001	0.004
PWTd (mm)	9 (1)	9 (1)	9 (0.5)	10 (1)	< 0.001	0.001
SV (ml)	68.5 (21.8)	68 (20)	61 (16)	79.5 (19)	< 0.001	0.001
EF (%)	68.31 ± 5.71	68.62 ± 5.85	68.61 ± 5.58	68.63 ± 6.41	0.992	0.583
Biochemical values						
Albumin (g/l)	41.1 (5.4)	41.1 (6.1)	41.9 (6.8)	39.8 (6)	0.19	0.287
Serum creatinine (μmol/l)	55.3 (15.2)	55.65(12.9)	54.5(12.1)	58 (18.2)	0.427	0.383
Total cholesterol (mmol/l)	3.36 (1.17)	3.5 (1.19)	3.71 (0.85)	3.21 (1.53)	0.208	0.299
Triglycerides (mmol/l)	0.9 (0.41)	0.93 (0.41)	0.88 (0.53)	0.99 (0.36)	0.15	0.256

Continuous variables are presented as the mean ± SD or the median (interquartile range) without a Gaussian distribution

## Results

### Subject characteristics

The proportion of females was higher than that of males in all the groups. On admission, 15 of the 82 patients and 11 of the 65 patients had undergone discontinuous medical treatment. Among these patients, 2 had simultaneously received intravenous iron treatment and blood transfusion, whereas others received oral or intravenous medication treatments. More details are shown in Table 1.

### Comparison of clinical and echocardiographic characteristics between patients with abnormal and normal LVMI

Comparison of data between IDA patients with normal and abnormal LVMI was performed to sift candidate parameters correlated to ventricular remodelling. The 65 patients enrolled in the hospital were divided into two groups according to abnormal LVMI status (> 115 g/m^2^ in men and > 95 g/m^2^ in women) [19]. The clinical and echocardiographic characteristics of all groups are shown in Table 1. The results show a significant upregulation of TIBC (*p* = 0.023) in the abnormal group but no statistically significant difference in parameters, including age, height, weight, BMI, HR, SBP, DBP, HCT, MCV, and EF compared with those in the control group. Additionally, we observe no discrepancy in serum iron, serum ferritin, TS or albumin between the two groups (*p* = 0.995, *p* = 0.422, *p* = 0.505, *p* = 0.19, respectively). Moreover, we find a higher duration of ID, higher RDW and lower haemoglobin levels in the abnormal LVMI group, but no statistically significant difference is found (*p* = 0.231, *p* = 0.09, *p* = 0.083, respectively).

### The correlation of LVMI with special parameters related to IDA

To further explore the correlation between continuous change of LVMI and other parameters, bivariate correlation and linear regression analysis were performed. Linear bivariate analysis with Pearson’s correlation coefficient reveal a positive correlation between log transformed LVMI and age (r Pearson’s = 0.293, *p* = 0.018), TIBC (r Pearson’s = 0.343, *p* = 0.005), and haemoglobin (r Pearson’s = − 0.276, *p* = 0.026), whereas there is no significant correlation between log transformed LVMI and BMI, HR, HCT, MCV, or EF (*p* = 0.341, *p* = 0.496, *p* = 0.092, *p* = 0.792, *p* = 0.148, respectively). Linear bivariate analysis with Spearman’s correlation coefficient reveal a significant correlation between LVMI and RDW (r Spearman’s = 0.243, *p* = 0.041) and SV (r Spearman’s = 0.702, *p* < 0.001). Moreover, a negative correlation is detected between LVMI and SBP, DBP, serum iron, serum ferritin, TS, and albumin (*p* = 0.933, *p* = 0.671, *p* = 0.703, *p* = 0.918, *p* = 0.426, *p* = 0.335, respectively).

**Fig. 1:**
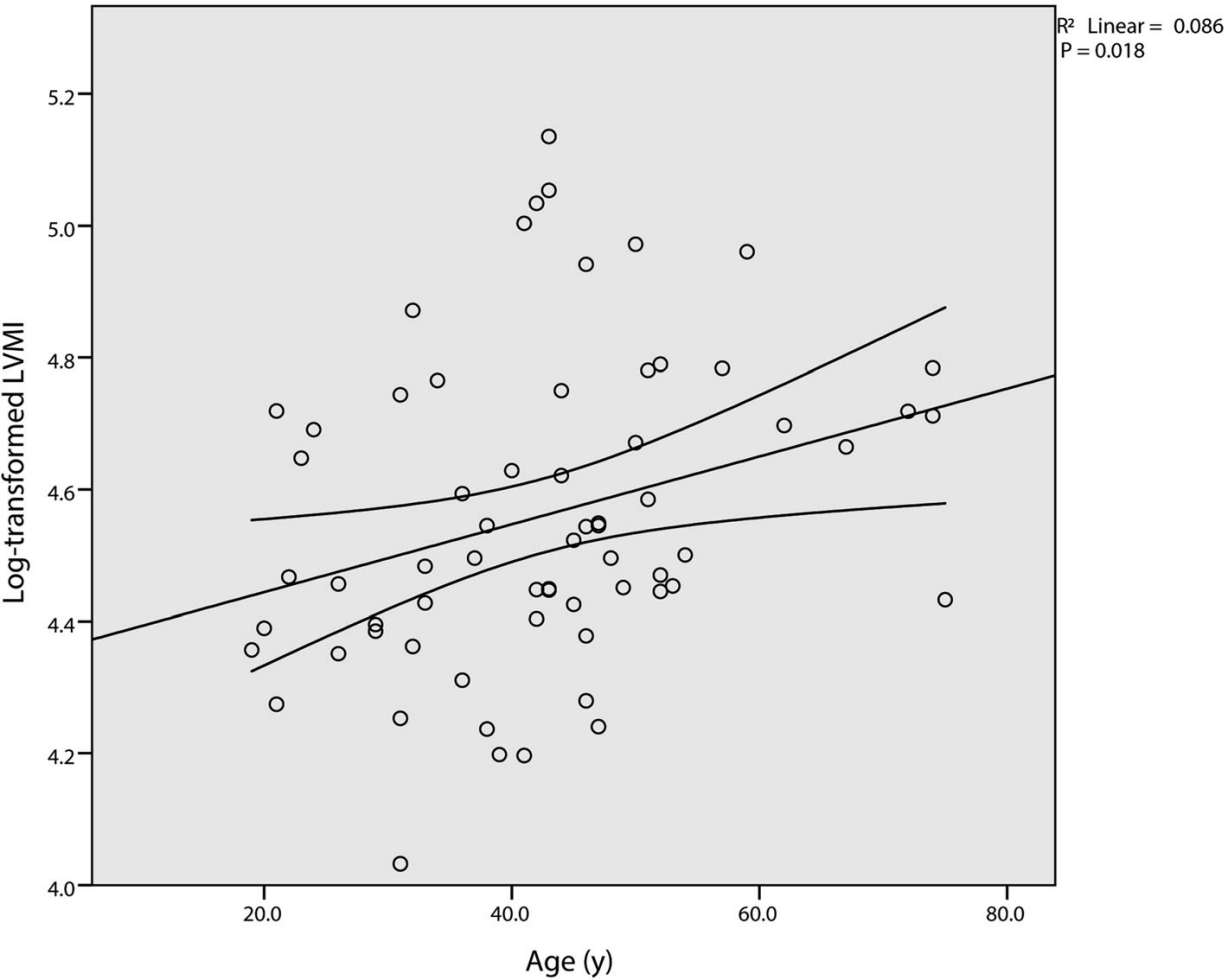
Simple scatterplot with regression line for age and log transformed LVMI. A positive correlation is depicted between the two variables. Curved lines represent 95% confidence intervals for the mean predicted log transformed LVMI

**Fig. 2:**
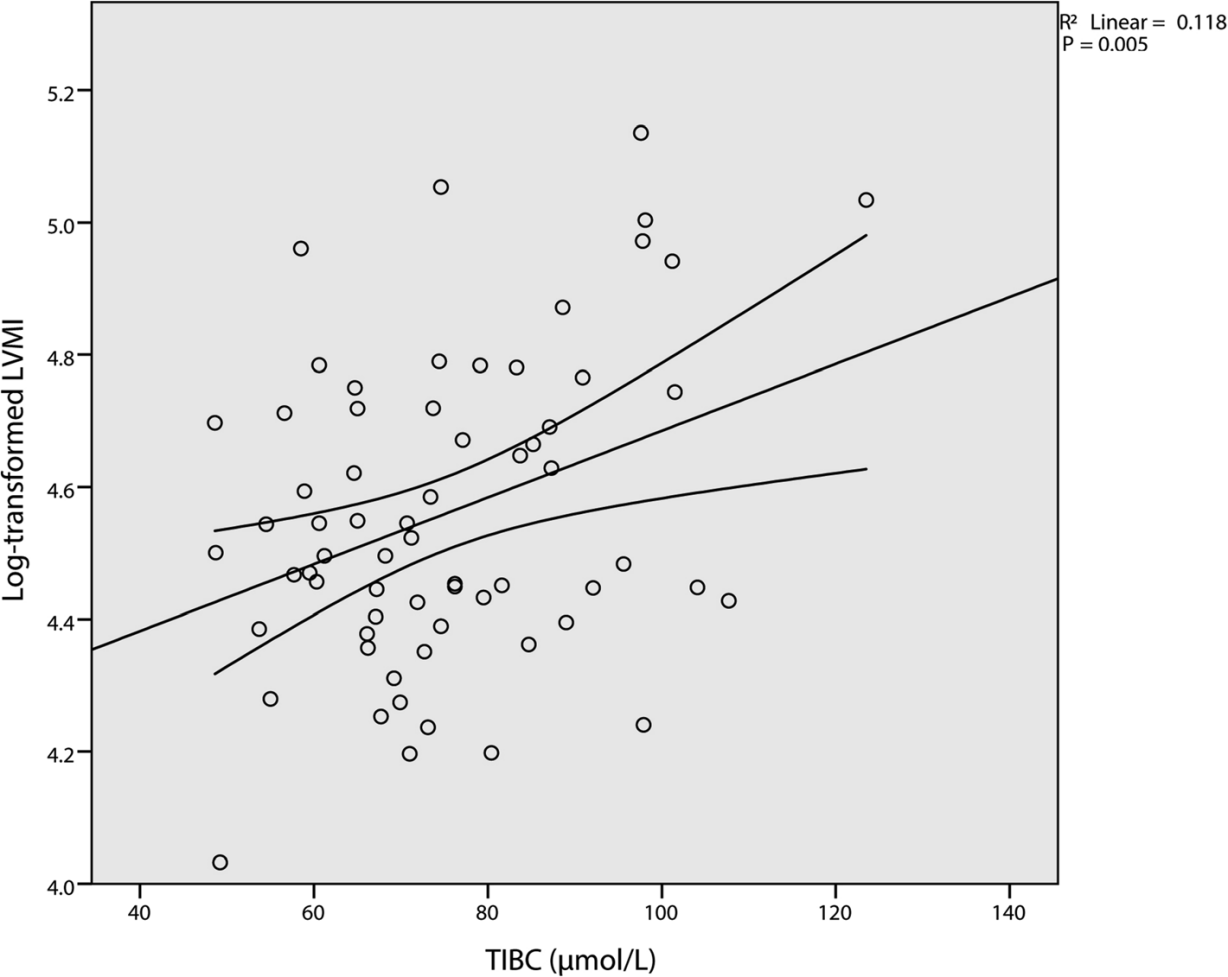
Simple scatterplot with regression line for total iron binding capacity (TIBC) and log transformed LVMI. A positive correlation is depicted between the two variables. Curved lines represent 95% confidence intervals for the mean predicted log transformed LVMI

**Fig. 3:**
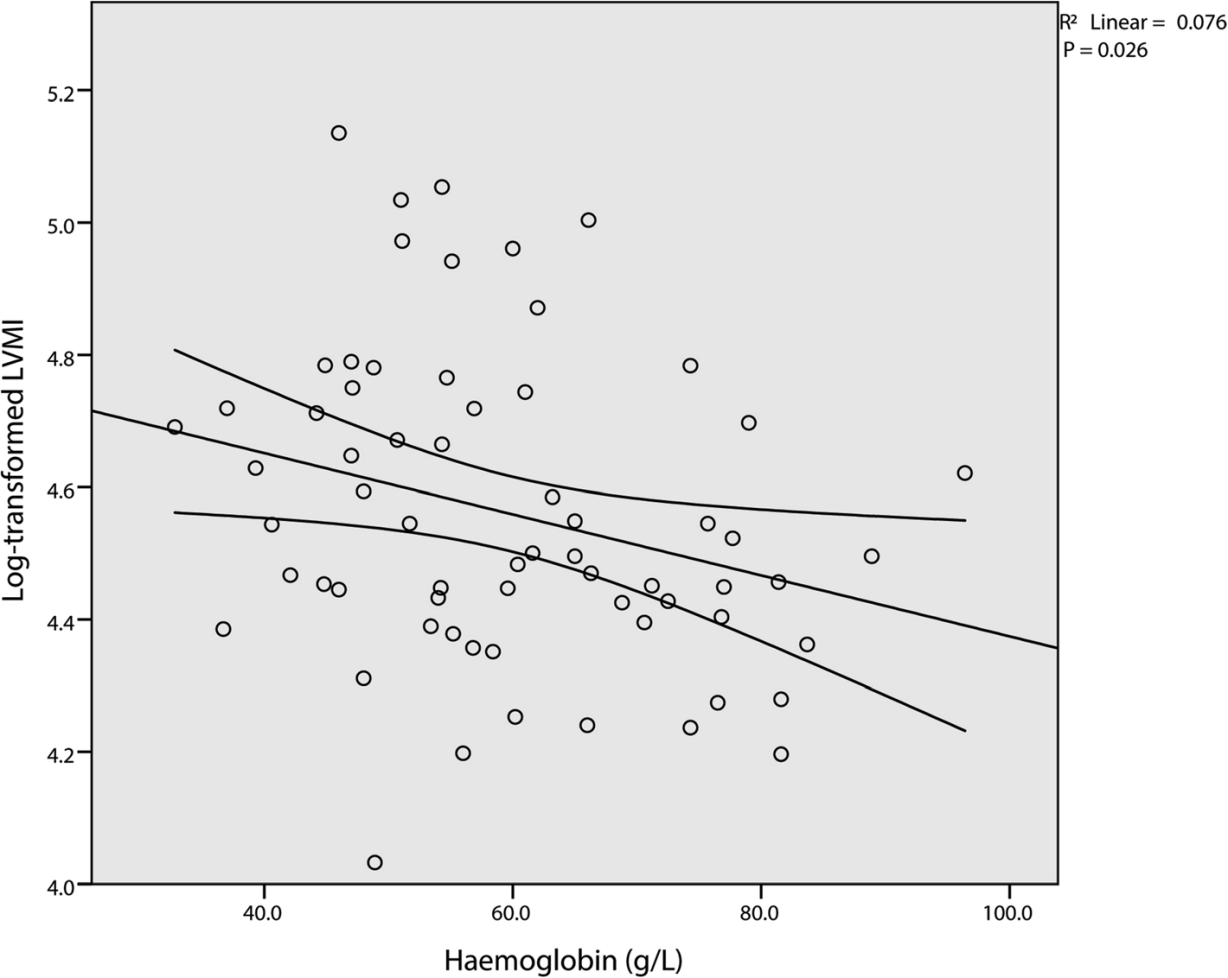
Simple scatterplot with regression line for haemoglobin and log transformed LVMI. A positive correlation is depicted between the two variables. Curved lines represent 95% confidence intervals for the mean predicted log transformed LVMI

Simple linear regression analysis is conducted by using log transformed LVMI as the dependent variable and other selected parameters as predictors (Table 2). Linear associations between log transformed LVMI and age (Fig. 1), TIBC (Fig. 2), and haemoglobin (Fig. 3) are verified. Yet only TIBC remain statistical significance after adjusted by Bonferroni correction. Multivariable linear regression analysis, which simultaneously introduced age (t = 3.269, *p* = 0.002), TIBC (t = 3.503, *p* = 0.001), and haemoglobin (t = − 2.459, *p* = 0.017) into the model, further confirm the validity of the associations (Table 3). Finally, we added the interaction term of TIBC and haemoglobin into the established model. The β of the interaction term was calculated to be zero, which denied the interaction between TIBC and haemoglobin.

**Table 2:** Simple linear regression analysis of log-transformed LVMI

	R	R^2^	Adjusted R^2^	B	P (Adjusted P)
Age	0.293	0.086	0.071	0.005	0.018 (0.054)
TIBC	0.343	0.118	0.104	0.005	0.005 (0.015)
Serum iron	0.067	0.004	−0.011	−0.006	0.597
Serum ferritin	0.057	0.003	− 0.015	− 0.003	0.673
TS	0.141	0.02	0.004	−0.009	0.261
Haemoglobin	0.276	0.076	0.061	−0.005	0.026 (0.078)
RDW	0.15	0.022	0.007	0.008	0.234
Albumin	0.062	0.004	−0.012	−0.002	0.63
HR	0.086	0.007	−0.008	−0.002	0.496
SBP	0.083	0.007	−0.009	0.002	0.511
DBP	0.054	0.003	−0.013	−0.002	0.67

Adjusted *P*-values were calculated by Bonferroni correction

**Table 3:** Multivariable linear regression analysis of log-transformed LVMI with age, haemoglobin and TIBC included in the model

Predictors	R^2^	Adjusted R^2^	B	SE of B	t	*p*
Age	0.305	0.271	0.006	0.002	3.269	0.002
TIBC			0.006	0.002	3.503	0.001
Haemoglobin			−0.004	0.002	−2.459	0.017

### The correlation of clinical and echocardiographic characteristics with haemoglobin or TIBC

Since both TIBC and haemoglobin were correlated to LVMI in our study, the multicollinearity and connection between them was necessary to take into consideration. We next explored the associations of haemoglobin or TIBC with clinical and echocardiographic characteristics in the 82 patients in order to reveal other parameters which may bridge the connection between them, for which a linear bivariate analysis was performed (listed in Table 4). Haemoglobin showed a positive relationship with parameters, including HR, RDW, serum iron, TS, LVDd, LVDs, and LVM. Moreover, TIBC exhibited a significant correlation with age, SBP, MCV, serum ferritin, TS, LVDd, LVM, SV and albumin. Results adjusted for multiple comparisons are showed in Table 4. Furthermore, we found no significant correlation between haemoglobin and TIBC (r Pearson’s = − 0.056, *p* = 0.658).

**Table 4:** Linear bivariate analysis of haemoglobin, TIBC and other parameters

	Haemoglobin		TIBC	
	r	P (Adjusted P)	r	P (Adjusted P)
Age	−0.055	0.622	−0.227	0.04 (0.36)
HCT	0.944	< 0.001(< 0.001)	−0.061	0.589
MCV	0.203	0.067	−0.319	0.003 (0.027)
RDW	−0.328	0.003 (0.024)	0.086	0.447
Serum iron	0.296	0.007 (0.056)	−0.114	0.307
Serum ferritin	0.19	0.105	−0.319	0.006 (0.054)
TS	0.289	0.009 (0.072)	−0.38	< 0.001 (0.003)
HR	−0.224	0.043 (0.344)	−0.005	0.964
SBP	0.173	0.12	0.357	0.001 (0.009)
DBP	0.126	0.26	0.172	0.122
LVM	−0.321	0.003 (0.024)	0.25	0.024 (0.216)
LVDd	−0.254	0.022 (0.176)	0.254	0.021 (0.189)
LVDs	−0.25	0.024 (0.192)	0.17	0.126
SV	−0.209	0.059	0.252	0.022 (0.198)
EF	0.125	0.263	−0.065	0.562
Albumin	0.163	0.149	0.359	0.001 (0.009)

Adjusted *P*-values were calculated by Bonferroni correction

### The relationships between LV remodelling, anaemia severity and TIBC

Finally, Logistic regression analysis was performed to determine the contribution of TIBC, anaemia severity, age, and duration of ID to the risk of LV remodelling. In the crude analysis, age, gender and ID duration fail to predict abnormal LVMI. However, TIBC (OR = 1.039, 95%CI = 1.001–1.070, *P* = 0.029) is significantly associated with the risk of LV remodelling. Though with *P* value greater than 0.05, the contribution of severe anaemia (haemoglobin < 60 g/L) to the risk of LV remodelling is nearly three times greater than that of non-severe anaemia (OR = 2.812, 95%CI = 0.962–8.224, *P* = 0.059). (Table 5).

**Table 5:** Logistic regression analysis of LV remodelling with anaemia severity and TIBC included in the predictive model

	Unadjusted OR	95% CI	*P*
Severe anaemia (yes or no)	2.812	0.962 to 8.224	0.059
TIBC (μmol/l)	1.039	1.001 to 1.070	0.029

## Discussion

The total number of binding sites for iron atoms on transferrin, TIBC is practically equivalent to the capacity of transferrin. The TIBC is one kind of ~ 80 kDa bilobal glycoprotein synthesized in and secreted by the liver. It is well known for its ability to deliver iron, [20] and the concentration of serum transferrin increases when ID occurs. As a proxy of transferrin, TIBC is not as subject to rapid changes in concentration as is plasma iron concentration, which is subject to considerable diurnal variation and influences of food intake, so TIBC is inherently more stable as an indicator of iron status [21]. Consequently, the day-to-day variations and assay variations in TIBC are low [22]. However, due to the large overlap between normal values and abnormal values in individuals with iron deficiency, abnormal TIBC levels merely reflect severe iron exhaustion and can only diagnose ID after the manifestation of anaemia [21]. Since all of the included patients in our study met the criteria of moderate-severe anaemia, TIBC should objectively reflect the extent of iron depletion in this study.

Ageing is an independent risk factor for the development of cardiovascular diseases, including HF and AF. The functional relationship between age and LVMI has also been firmly established [23–25]. While emerging evidence verified a correlation between TIBC and age, it seems possible that the association between TIBC and LVMI reflect the age-LVMI interrelation. However, the inverse correlation between TIBC and age made this speculation difficult to explain. In addition, no multi-collinearity in the multivariate regression model was found when both age and TIBC were included.

TIBC has acted as a surrogate nutritional markers in multiple studies, [26, 27] and lower TIBC predicted poorer prognosis in both postoperative and haemodialysis patients [28, 29]..

The positive correlation found between albumin and TIBC in our study adequately corroborates the nutritional significance of TIBC. Nevertheless, we found a tendency towards increased TIBC in the larger LVMI group, which contradicts the inclination that TIBC should have in reflecting malnourishment-related cardiomyopathy. Normal albumin values and a lack of statistical relation to LVMI in the linear bivariate analysis further eliminated the possible nutritional influences that TIBC could have on LV. Notably, oral contraceptive (OC) use can markedly increase TIBC [30]. However, the unexpected rise in TIBC mainly affects its representativeness of ID. In our study, TIBC is still strongly correlated with serum ferritin according to our statistical results. Apart from this, perimenopause women made up the majority of the population included in our study, which minimized the discrepancy that OC use could cause.

Circulating ferritin was consistently not correlated with either LVMI in the 65 patients or SV in the 82 patients in this study. The serum ferritin assay has been widely used as the most common method to evaluate body iron storage. However, in contrast to the soluble transferrin receptor (sTfR), which is sensitive to erythropoiesis and low tissue iron utilization regardless of iron stores, ferritin is characterized as a “storage parameter” rather than a “functional parameter” [31]. Serving as a ligand of sTfR, it is presumable that transferrin may perform superiorly to ferritin in the evaluation of iron usage in the myocardium.

In a study of 298 postoperative patients also found correlations between age, albumin and TIBC [29]. Interestingly, both our current study and previous one consistently exhibit no significant association of TIBC with haemoglobin level. Associations underlying them are historically perplexing. In the past decades, less progress has been made among studies trying to use iron indictors as predictors of the response of the haemoglobin concentration to iron treatment [21]. Exploration of associations between haemoglobin, TIBC and baseline clinical and echocardiographic characteristics was performed within the 82 initially enrolled patients. However, none of the parameters included in our research, except for TS, were simultaneously associated with both haemoglobin and TIBC. Recent evidence show that erythroferrone (ERFE), a hepcidin suppressors, [32] are both related to TIBC and haemoglobin, [33] which suggested haematopoietic regulation, which links both anaemia and iron metabolism, needs to be taken into account. However, such a correlation does not always seem to exist. By assaying erythropoietin in 136 pregnant women, Carretti and colleagues found that variations in EPO were closely correlated with transferrin throughout the whole pregnancy period, while serum haemoglobin concentration and serum iron seemed to not always correlated with EPO [34].

The obvious flaw in our study is the limited number of patients enrolled. However, owing to the consequent minimization of discrepancies and the compact model, apparent correlations could be identified. The intrinsic retrospective nature also requires the conclusions of our study to be interpreted cautiously. Hence, a rational discussion based on our evidence seems to be crucial. Also due to the small sample size, interaction test and logistic regression may be underpowered in our study. Since LVMI changes in continuous way, arbitrarily dichotomize it by cut off value may inevitable lead to some grouping inaccuracy and data wastage. Additionally, we failed to determine the impact that the duration of ID may have on the remodelling process. Since we must admit the subjective uncertainty resulting from our data acquisition method, further prospective research is warranted. Most patients in our study are middle age female with minor symptoms and less complications. In that way the scope of inference of this study is likely inclined to such population group. Finally, observer variability in TTE findings should be mentioned.

## Conclusion

Results of study indicate that TIBC exhibit an explicit association with LVMI in patients with iron deficiency anaemia. Yet due to the small sample size our study applies, the potential relationship between other traditional iron indicators included in study should not be arbitrarily denied. Logistic analysis further confirms the contribution of TIBC to abnormal LVMI incidence among this population with IDA. Owing to the critical position that iron occupies in myocardial biological metabolism and its emerging prognostic value among patients with HF, further large-scale investigations of the relationship between ID and cardiac remodelling are urgently needed.

## Data Availability

The datasets used and/or analysed during the current study are available from the corresponding author on reasonable request.

## Abbreviations

CKD: Chronic kidney disease
DBP: Diastolic blood pressure
EF: Ejection fractions
HCT: Haematocrit
HF: Heart failure
HGB: Haemoglobin
HR: Heart rate
ID: Iron deficiency
IDA: Iron deficiency anaemia
IVSTd: Interventricular septum diastolic diameters
LVDd: Left ventricular end-diastolic dimensions
LVDs: End-systolic dimensions
LVPWT: Left ventricular posterior wall thicknesses
MCV: Erythrocyte mean corpuscular volume
RDW: Red blood cell distribution width
SBP: Systolic blood pressure
SCD: Sickle cell disease
SV: Stroke volume
TIBC: Total iron binding capacity
TS: Transferrin saturation
